# A Potential Diagnostic Approach for Foetal Long-QT Syndrome, Developed and Validated in Children

**DOI:** 10.1007/s00246-018-1911-y

**Published:** 2018-05-22

**Authors:** Arja Suzanne Vink, Irene M. Kuipers, Rianne H. A. C. M. De Bruin-Bon, Arthur A. M. Wilde, Nico A. Blom, Sally-Ann B. Clur

**Affiliations:** 1 Heart Centre, Department of Cardiology, Academic Medical Centre, University of Amsterdam, PO Box 22660, 1100 DD Amsterdam, The Netherlands; 2 Department of Paediatric Cardiology, Emma Children’s Hospital, Academic Medical Centre, Amsterdam, The Netherlands; 3 Department of Paediatric Cardiology, Willem-Alexander Children’s Hospital, University Medical Centre Leiden, Leiden, The Netherlands

**Keywords:** Paediatrics, Echocardiography, Electrocardiogram (ECG), Long-QT syndrome, Foetus

## Abstract

In patients with Long-QT Syndrome (LQTS), mechanical abnormalities have been described. Recognition of these abnormalities could potentially be used in the diagnosis of LQTS, especially in the foetus where an ECG is not available and DNA-analysis is invasive. We aimed to develop and validate a marker for these mechanical abnormalities in children and to test its feasibility in foetuses as a proof of principle. We measured the myocardial contraction duration using colour Tissue Doppler Imaging (cTDI) in 41 LQTS children and age- and gender-matched controls. Children were chosen to develop and validate the measurement of the myocardial contraction duration, due to the availability of a simultaneously recorded ECG. Feasibility of this measurement in foetuses was tested in an additional pilot study among seven LQTS foetuses and eight controls. LQTS children had a longer myocardial contraction duration compared to controls, while there was no statistical difference in heart rate. Measuring the myocardial contraction duration in children had a high inter- and intra-observer validity and reliably correlated with the QT-interval. There was an area under the curve (AUC) of 0.71, and the optimal cut-off value showed an especially high specificity in diagnosing LQTS. Measuring the myocardial contraction duration was possible in all foetuses and had a high inter- and intra-observer validity (ICC = 0.71 and ICC = 0.88, respectively). LQTS foetuses seemed to have a longer myocardial contraction duration compared to controls. Therefore, a prolonged contraction duration may be a potential marker for the prenatal diagnosis of LQTS in the future. Further studies are required to support the measurement of the myocardial contraction duration as a diagnostic approach for foetal LQTS.

**Electronic supplementary material:**

The online version of this article (10.1007/s00246-018-1911-y) contains supplementary material, which is available to authorized users.

## Introduction

Congenital Long-QT syndrome (LQTS) in an inherited cardiac repolarization disorder, with a predisposition to malignant ventricular arrhythmias, that can precipitate syncope, sudden cardiac arrest or sudden cardiac death [1]. The degree of impairment and dispersion of repolarization have been considered to play a role in the occurrence of these ventricular arrhythmias [2]. Although, patients with LQTS have a normal left ventricular function, several studies have shown mechanical abnormalities in the contraction and relaxation pattern in adult LQTS patients using M-Mode echocardiography,[3–5] continuous-wave Doppler images,[6] colour Tissue Doppler Imaging (cTDI) [7, 8] and Speckle Tracking [9–11]. One study has found mechanical dysfunction in children with the use of MRI [12]. These abnormalities were almost absent in healthy individuals, but prevalent among LQTS patients whereas in symptomatic patients this was even more frequent, suggesting their potential value in the diagnosis and risk stratification in LQTS.

The use of the presence of mechanical abnormalities in the risk stratification for malignant cardiac arrhythmias in adult and paediatric LQTS patients is perhaps more promising than its use in diagnosing LQTS, since most LQTS patients can be diagnosed based on a high clinical suspicion using electrocardiographic features, clinical and family history or in the presence of a confirmed pathogenic mutation [13]. Diagnosing LQTS prenatally is however challenging due to the lack of an accurate and comprehensible electrocardiogram (ECG), [14] the scarcity of foetal magnetocardiography, [15–18] and the invasiveness of DNA-analysis with a risk of miscarriage [19]. To date, foetal arrhythmias are diagnosed by echocardiography providing accurate information about the atrial and ventricular contractions that indirectly reflect the P-wave and QRS-complex [20]. Unfortunately echocardiography cannot assess a derivative of the QT-interval.

However, diagnosing LQTS before birth is relevant as it is a cause of sudden infant death and sudden unexplained intrauterine death [14, 21, 22]. In addition, when LQTS is diagnosed during foetal life there are important clinical consequences [14]. For instance, maternal QT-prolonging drugs frequently used during pregnancy and childbirth must be withheld, even if the mother does not suffer from LQTS. Furthermore, in case of foetal arrhythmias, some antiarrhythmic drugs that are usually given are contraindicated if the foetus has LQTS, e.g. amiodarone [23–25]. Lastly, a sinus bradycardia, 2:1 atrioventricular conduction or reduced beat to beat variability in the foetus can be attributed to the LQTS phenotype [26] instead of foetal distress. Hence, an accurate prenatal diagnosis of LQTS can avoid potentially unnecessary preterm deliveries or emergency caesarean sections for incorrectly presumed foetal distress.

Haugaa et al. [8] used a combination of cTDI and a simultaneously recorded ECG to evaluate the mechanical abnormalities in adult LQTS patients, and found a prolonged myocardial contraction duration in these patients. As cTDI has proven to be feasible in foetuses, has already been used in the evaluation of foetal cardiac function in various pregnancy complications,[27–29] and has been shown to be reliable in the measurement of foetal time intervals,[30] it could potentially be used for the measurement of the myocardial contraction duration prenatally. However, this measurement then needs to be made in the absence of an ECG.

This study had three aims: Firstly, to assess the diagnostic value of measuring the myocardial contraction duration by measuring it in children and correlating it to the QT-interval on the ECG. Children were chosen due to the availability of a simultaneously recorded ECG. Secondly, we aimed to develop a technique for the measurement of the myocardial contraction duration without the need for a simultaneously recorded ECG in children and to test its diagnostic value. Lastly, we wished to test the feasibility of this developed technique of measuring the myocardial contraction duration without a simultaneously recorded ECG in a pilot study among foetuses as a proof of principle.

## Methods

### Study Population (Children)

This cross-sectional study included genotype-positive LQTS children under the age of 18 years, and age- and gender-matched controls. LQTS was diagnosed based on a confirmed pathogenic mutation detected using conventional methods. Controls were referred for cardiac screening and a normal heart was confirmed after a clinical examination, ECG and echocardiography. The ECG and echocardiogram of the cases were performed during a regular follow-up visit and of the controls during a screening visit at the outpatient clinic of our hospital. The study was approved by the institutional Review Board.

### Electrocardiogram (Children)

A 12-lead ECG was obtained in all subjects, either straight before or after echocardiography. All ECGs were digitalized, blinded and manually analysed by one investigator (ASV) using Image J 1.50i [National Institutes of Health, USA]. QT-intervals of three consecutive complexes were measured using the tangent method (QT-12ECG). Preceding RR-intervals were obtained (RR-12ECG), and the QTc-interval was calculated using the Bazett’s correction formula,[31] after the three consecutive complexes were averaged (QTc-12ECG).

### Echocardiography (Children)

**Fig. 1:**
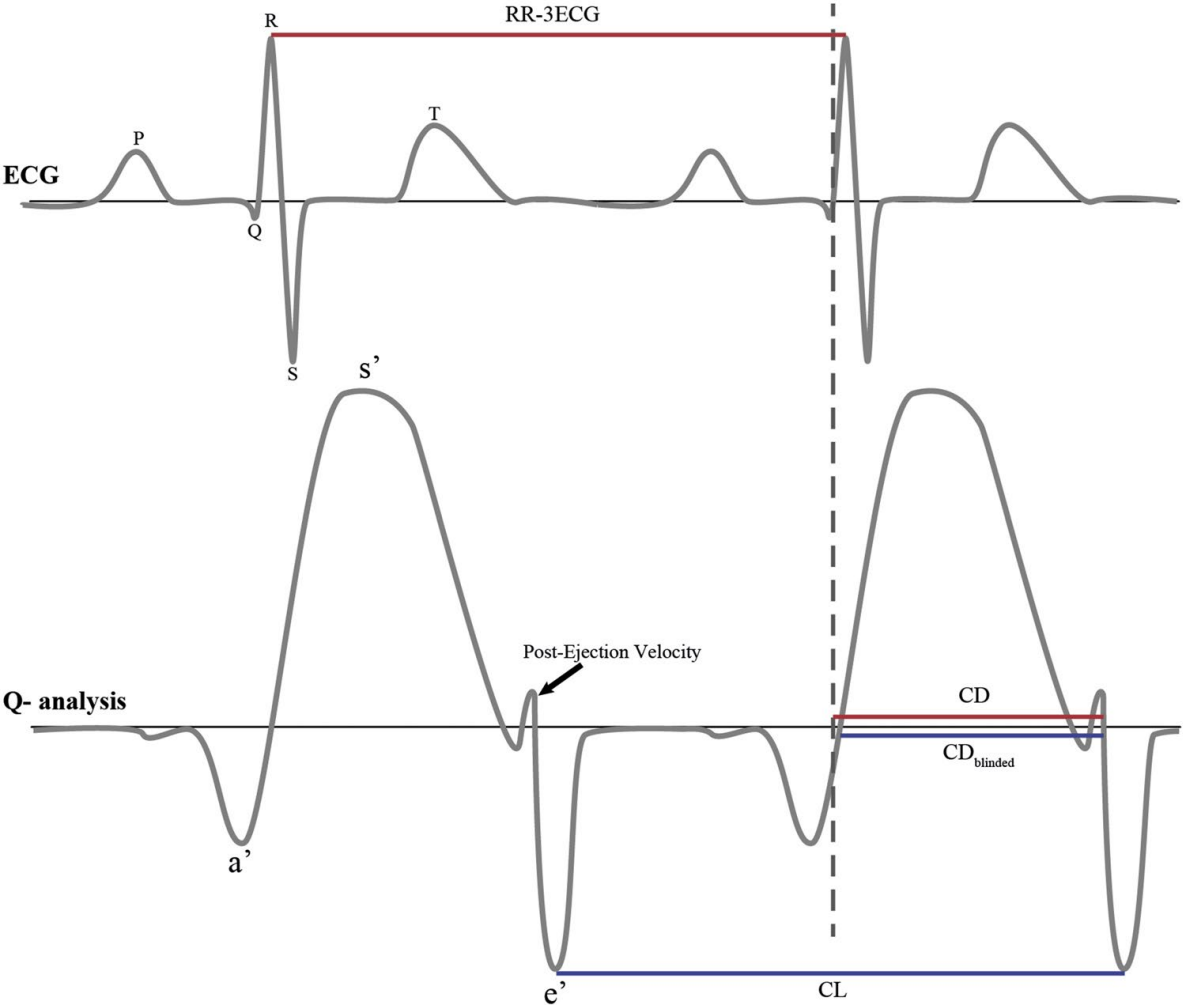
Schematic figure of the echocardiography parameters. Red parameters were measured unblinded for the 3-lead ECG, blue parameters were measured blinded for the 3-lead ECG. *RR-3ECG* RR-interval on the 3-lead ECG throughout cTDI recording, *CD* myocardial contraction duration measured unblinded for the surface ECG, *CD*_*blinded*_ myocardial contraction duration measured blinded for the surface ECG, *CL* cycle length. *s*′ first-peak systolic velocity of the annulus, *e′* early peak diastolic velocity, *a′* late peak diastolic velocity

In all subjects an echocardiogram was obtained using Vivid 7 [GE, Healthcare, Horten, Norway]. The shortening fraction (SF) was calculated from the left ventricle end-diastolic and end-systolic diameters measured on M-mode. In addition, a two-dimensional guided cTDI recording was obtained by well-trained echocardiographers. All cTDI images were obtained with a frame rate of ≥ 95 frames per second from an apical four chamber view and analysed using commercially offline available software [Q-Analysis, EchoPac®, GE] averaging three cycles with the cursor on the basal interventricular septum (IVS). The IVS was chosen because of its relative easy determination in the small foetal heart. The following parameters were assessed (Fig. 1):

I. The myocardial contraction duration (CD) as described by Haugaa et al. [8], defined as the time from start of R on the 3-lead ECG that was recorded throughout the cTDI procedure, to the end of the post-ejection velocity (PEV) at zero-crossing or to zero-crossing of the decreasing velocity slope in end-systole when no positive PEV was present.
II. RR-intervals on the 3-lead ECG (RR-3ECG) recorded throughout the cTDI procedure.
III. The myocardial contraction duration blinded for the 3-lead ECG recorded throughout the cTDI procedure (CD_blinded_), defined as the time from the first positive velocity slope before peak ejection velocity (zero-crossing), to the end of the PEV at zero-crossing or to zero-crossing of the decreasing velocity slope in end-systole when no positive PEV was present.
IV. Cycle length (CL), defined as the time between the two successive troughs of the velocity slope in end-systole, including the CD_blinded_.

All parameters were measured blinded for subjects’ characteristics. It was ensured that there was sufficient time between the parameters unblinded (CD and RR-3ECG) and blinded for the 3-lead ECG recording throughout the cTDI procedure (CD_blinded_ and CL) in order to avoid recall. One observer (ASV) measured all the parameters and these measurements were used in the primary analyses. A second observer (SC) measured the CD_blinded_ and CL to obtain inter-observer validity and the CD, CD_blinded_ and CL were re-measured by ASV in order to obtain intra-observer validity.

### Pilot Study (Foetuses)

The feasibility of measuring the CD_blinded_ and CL in foetuses was tested in a pilot study among foetuses with paternal or maternal genotype-positive LQTS, and healthy foetuses with a normal heart on the echocardiogram. LQTS was diagnosed after birth if the family mutation was found.

In all foetuses a two-dimensional guided cTDI recording was obtained by one foetal cardiologist (SC). The angle of insonation of the long axis of the heart was kept as small as possible, and recordings were made in the absence of foetal movement. Images were digitally saved with a frame rate of ≥ 130 frames per second. The cTDI recordings were analysed using Q-Analysis [EchoPac®, GE] averaging three cycles with the circular marker (diameter 3 mm) on the basal  IVS. The CD_blinded_ and CL were measured blinded for subjects’ characteristics by two observers (ASV and SC) to obtain inter-observer validity and a repeated analysis was done only by ASV a week later to obtain intra-observer validity.

### Statistical Analysis

Clinical and echocardiographic characteristics were presented as frequencies (percentage) for categorical variables, mean (95% confidence intervals; CI) for continuous variables with an approximately symmetrical distribution and median (interquartiles) for continuous data with a skewed distribution. Differences between LQTS children and controls were analysed as appropriate by a paired t-test or a Wilcoxon signed-rank test for continuous data, and by a McNemar’s test for binary data. Differences between LQTS foetuses and controls were only presented as an absolute mean difference based on either all the first or all the last echocardiograms of the foetuses. In children, a multiple linear regression was performed to correct the CD and CD_blinded_ for the heart rate (i.e. RR-3ECG and CL, respectively) stratified by LQTS patients or controls.

Inter-method variability in children was defined between 12-lead ECG and echocardiographic parameters, as well as between the CD and CD_blinded_. The inter-method variability in children and the intra- and inter-observer validity for both children and foetuses were all expressed as correlation coefficients estimated by a Pearson correlation or Spearman rank correlation test as appropriate, and the intra-class correlation coefficient (ICC) for multiple measurements based on a consistency (inter-method variability and intra-observer validity) and two-way agreement (inter-observer validity) model according to Ciccheti [32] and Fleiss [33]. Bland–Altman analyses [34] were performed to assess bias and limits of agreement for the inter-method in children and the intra- and inter-observer validity for both the children and the foetuses.

Receiver-operating characteristic (ROC) curves were constructed to determine the area under the curve (AUC) and the sensitivity and specificity for specific cut-off values of the QT-12ECG, QTc-12ECG, CD and CD_blinded_ to identify LQTS children. The cut-off value of the QTc-interval was established at 460 ms [35] and the optimal cut-off value for both the CD and CD_blinded_, was defined as the Youden’s index. The reliability of the cut-off values was validated using bootstrap resampling (*n* = 1.000) [36].

Sampling uncertainty was quantified via 95% confidence intervals (CI) and *P* values. A *P* value < 0.05 was considered to be statistically significant. Data were analysed with R version 3.3.2 [R Foundation for Statistical Computing, Vienna, Austria].

## Results

### Children’s Characteristics

cTDI recordings were performed in 160 eligible subjects until April 2017. Forty-one LQTS patients could appropriately be matched to controls with respect to age and gender (Table 1). The QTc-interval was prolonged in LQTS patients (*P* < 0.001) with an absolute mean difference of 44 ms compared to the controls. LQTS patients were mainly diagnosed as a result of family screening (88%). There were five symptomatic LQTS patients, of whom only one had a QTc-interval > 460 ms at the time of the cTDI recording during follow-up. Of these five symptomatic patients, three patients were symptomatic before diagnosis. One had a syncopal event after waking up from a loud noise, 4 years before the data used in this study was obtained. Another patient had a syncope during cycling, within the same year as the collected ECG and echocardiogram in this study. Another patient had an event of near-drowning 2 years before the included ECG and echocardiogram. Two patients had a cardiac event after diagnosis. One patient had (pre)syncopal episodes after a nightmare, one year after our included data. In addition, there was one LQTS type 3 patient that had a sudden cardiac death during follow-up while he was treated with propranolol and mexiletine. A pacemaker and a subcutaneous implantable cardioverter defibrillator had to be explanted due to endocarditis and persistent furunculosis. The included ECG and echocardiogram were made 4 years before his death. Four asymptomatic LQTS patients had a QTc-interval > 460 ms. In all the included subjects, there was a normal SF with no difference between the LQTS patients and controls (*P* = 0.640). The genotypes of the LQTS patients are shown in Supplementary Table 1.

**Table 1:** Clinical characteristics and echocardiographic parameters of the included children

	LQTS *n* = 41	Controls *n* = 41	*P* value
*Clinical characteristics*			
Age, years (interquartiles)	11 (6–14)	11 (7–14)	0.775
Female, *n* (%)	15 (37)	15 (37)	0.118
RR-12ECG, ms (95% CI)	906 (838–974)	818 (760–876)	0.104
QT-12ECG, ms (95% CI)	407 (389–425)	347 (335–358)	< 0.001
QTc-12ECG, ms (95% CI)	431 (422–441)	387 (381–392)	< 0.001
SF, % (95% CI)	39 (39–41)	40 (39–42)	0.640
Beta-blocker therapy, *n*_(%)_	34 (83)	0 (0)	0.023
*Echocardiographic parameters*			
CD, ms (95% CI)	420 (403–438)	383 (367–399)	0.005
RR-3ECG, ms (95% CI)	927 (855–999)	837 (768–905)	0.130
CD_blinded_, ms (95% CI)	412 (397–428)	373 (356–391)	0.004
CL, ms (95% CI)	927 (854–999)	836 (767–904)	0.126

*RR-12ECG* RR-interval on the 12-lead ECG, *ms* milliseconds, *CI* confidence interval, *QT-12ECG* QT-interval on the 12-lead ECG, *QTc-12ECG* QTc-interval on the 12-lead ECG, *SF* shortening fraction, *CD* myocardial contraction duration measured unblinded for the surface ECG, *RR-3ECG* RR-interval on the 3-lead ECG throughout cTDI recording, *CD*_*blinded*_ myocardial contraction duration measured blinded for the surface ECG, *CL* cycle length measured blinded for the surface ECG.

### Contraction Duration in Children

The CD and CD_blinded_ were both significantly prolonged in LQTS patients compared to controls, with an absolute mean difference of 37 ms (*P* = 0.005) and 39 ms (*P* = 0.004), respectively (Table 1). Though not statistically significant, LQTS patients had a longer RR-3ECG and CL compared to controls. However, after correction for RR-3ECG by a multiple linear regression analysis, the CD remained prolonged in LQTS patients compared to controls (*P* = 0.011). The same was found when the CD_blinded_ was corrected for CL (*P* = 0.006).

### Inter-method Variability in Children

Inter-method variability between the 12-lead ECG and echocardiographic parameters, and between the two echocardiographic parameters CD and CD_blinded_, are shown in Table 2. There was a high consistency between the RR-12ECG and RR-3ECG (ICC = 0.92, *P* < 0.001), indicating no major differences in heart rate between the 12-lead ECG and echocardiogram. In addition, there was a strong agreement between the QT-12ECG and CD (ICC = 0.87, *P* < 0.001). Inter-method variability between the CD_blinded_ and the 12-lead ECG parameters showed similar results as the CD.

**Table 2:** Correlation and Intra-class correlation coefficients (ICC) and Bland–Altman analyses in children

	Correlation coefficient (95% CI)	*P* value	ICC (95% CI)	*P* value	Mean difference in ms (± Limits of agreement in ms)
*12-lead ECG vs Echocardiographic parameters*					
RR-12ECG vs RR-3ECG	0.85 (0.78–0.90)	< 0.001	0.92 (0.87–0.95)	< 0.001	− 20 (± 240)
QT-12ECG vs CD	0.77 (0.67–0.85)	< 0.001	0.87 (0.80–0.92)	< 0.001	− 25 (± 76)
QTc-12ECG vs CD	0.36 (0.16–0.54)	< 0.001	0.45 (0.15–0.65)	0.004	7 (± 111)
QT-12ECG vs CD_blinded_	0.67 (0.53–0.77)	< 0.001	0.80 (0.69–0.87)	< 0.001	− 16 (± 91)
QTc-12ECG vs CD_blinded_	0.28 (0.07–0.47)	0.009	0.38 (0.03–0.60)	0.017	16 (± 114)
*Echocardiographic parameters*					
CD vs CD_blinded_	0.77 (0.66–0.84)	< 0.001	0.87 (0.80–0.92)	< 0.001	9 (± 77)
*Intra-observer validity*					
CD	0.92 (0.87–0.95)	< 0.001	0.96 (0.93–0.97)	< 0.001	− 8 (± 46)
CD_blinded_	0.89 (0.83–0.93)	< 0.001	0.94 (0.91–0.97)	< 0.001	1 (± 52)
CL	1.00 (1.00–1.00)	< 0.001	1.00 (1.00–1.00)	< 0.001	− 1 (± 28)
*Inter-observer validity*					
CD_blinded_	0.84 (0.77–0.90)	< 0.001	0.91 (0.84–0.94)	< 0.001	11 (± 62)
CL	1.00 (1.00–1.00)	< 0.001	1.00 (1.00–1.00)	< 0.001	1 (± 21)

*CI* confidence interval, *ms* milliseconds, *RR-12ECG* RR-interval on the 12-lead ECG, *RR-3ECG* RR-interval on the 3-lead ECG throughout cTDI recording, *QT-12ECG* QT-interval on the 12-lead ECG, *CD* myocardial contraction duration measured unblinded for the surface ECG, *QTc-12ECG* QTc-interval on the 12-lead ECG, *CD*_*blinded*_ myocardial contraction duration measured blinded for the surface ECG, *CL* cycle length measured blinded for the surface ECG

**Fig. 2:**
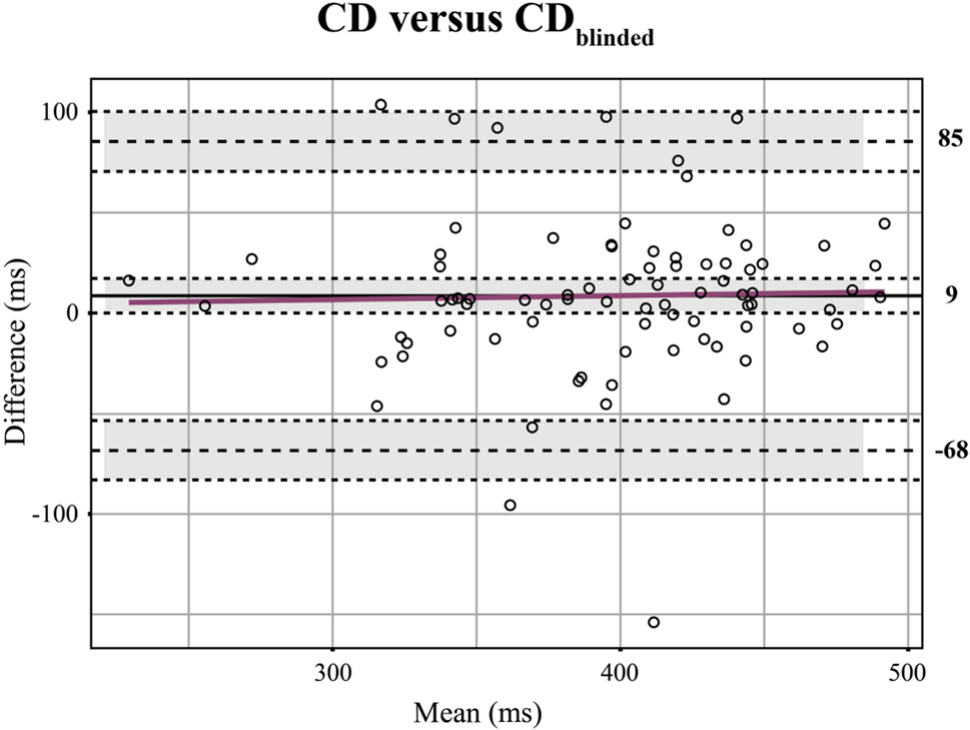
Bland–Altman plot for the comparison of CD and CD_blinded_ in children. In grey the 95% confidence interval around the mean and the limits of agreement. In pink the regression lines. *ms* milliseconds

Comparing the two echocardiographic parameters, CD and CD_blinded_, showed a high consistency (ICC = 0.87, *P* < 0.001) with a systematic error of 9 ms and a maximal difference of 77 ms. The Bland–Altman plot is shown in Fig. 2.

### Intra-and Inter-observer Validity in Children

The intra-observer validity was very high for measuring the CD (ICC = 0.96, *P* < 0.001) with a systematic error of 8 ms (maximal difference 46 ms). The reproducibility for the CD_blinded_ had approximately the same magnitude as the CD (ICC = 0.94, *P* < 0.001) with a smaller systematic error (1 ms with a maximal difference of 52 ms). The inter-observer validity for CD_blinded_ was also high (ICC = 0.91, *P* < 0.001). The intra- and inter-observer validity for CL was very high (for both ICC = 1.00 and *P* < 0.001) with for the intra-observer validity a systematic error of 1 ms (maximal difference 28 ms) and for the inter-observer validity a systematic error of 1 ms (maximal difference 21 ms).

### Sensitivity and Specificity in Children

**Fig. 3:**
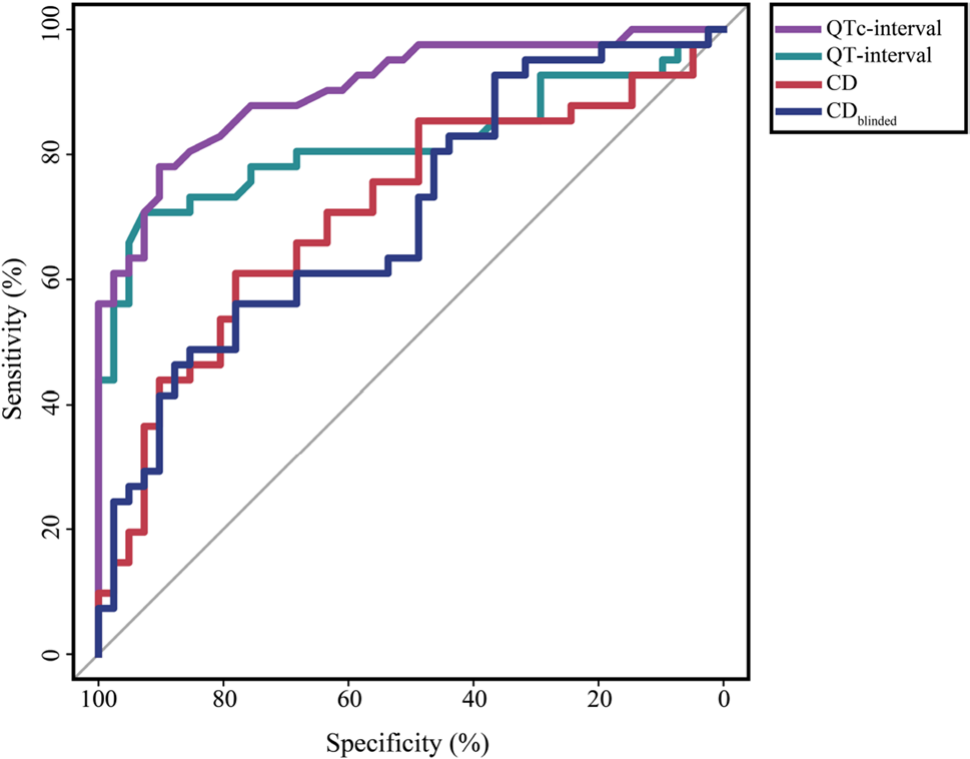
Receiver-operating characteristic curves of the diagnosis LQTS in children for the QT-interval, QTc-interval, CD and CD_blinded_

There was an AUC of 0.71 (95% CI by bootstrapping 0.47–0.71) for the CD and the optimal cut-off value of 422 ms (95% CI by bootstrapping 371–448 ms) yielded a sensitivity of 61% (95% CI by bootstrapping 46–76%) and a specificity of 78% (95% CI by bootstrapping 63–90%) for the diagnosis of LQTS. The CD_blinded_ had a similar distinctiveness compared to the CD. In this relatively asymptomatic group of LQTS patients, the optimal cut-off value for the CD and CD_blinded_ yielded a sensitivity of approximately 60% and a specificity of 78% (Table 3). In Fig. 3 the ROC curves are shown for the QT-12ECG, QTc-12ECG, CD and CD_blinded_.

**Table 3:** Area under the curve (AUC), cut-off values with corresponding sensitivity and specificity in children

	AUC (95% CI)	Cut-off value (95% CI)	Sensitivity (95% CI)	Specificity (95% CI)
QT-12ECG	0.82 (0.50–0.77)	460 ms (NA)	17% (7–29)	100% (100–100)
QTc-12ECG	0.91 (0.66–0.91)	460 ms (NA)	12% (2–22)	100% (100–100)
CD	0.71 (0.47–0.71)	422 ms (371–448)	61% (46–76)	78% (63–90)
CD_blinded_	0.71 (0.53–0.72)	415 ms (338–442)	56% (41–71)	78% (66–90)

*CI* confidence interval, *QT-12ECG* QT-interval on the 12-lead ECG, *QTc-12ECG* QTc-interval on the 12-lead ECG, *CD* myocardial contraction duration measured unblinded for the surface ECG, *CD*_*blinded*_ myocardial contraction duration measured blinded for the surface ECG, *ms* milliseconds, *NA* not applicable

### Pilot Study in Foetuses

Fifteen foetuses (*n* = 7 LQTS and *n* = 8 controls) were included in the pilot study with a total of 24 echocardiograms (Supplementary Table 2). The CD_blinded_ and CL could be determined in all cTDI recordings by both observers. The intra-observer validity for the determination of the CD_blinded_ was high (*r* = 0.79 and ICC = 0.88 both *P* < 0.001) with a systematic error of 4 ms (maximal difference 69 ms) and the inter-observer validity was good (rho = 0.68, *P* < 0.001 and ICC = 0.71, *P* = 0.002) with a systematic error of 16 ms (maximal difference 87 ms). For the CL, the intra-observer validity was very high (*r* = 0.87 and ICC = 0.93 both *P* < 0.001) with a systematic error of 8 ms (maximal difference 45 ms) and the inter-observer validity was high (*r* = 0.74 and ICC = 0.84 both *P* < 0.001) with a systematic error of 11 ms (maximal difference 68 ms).

**Fig. 4:**
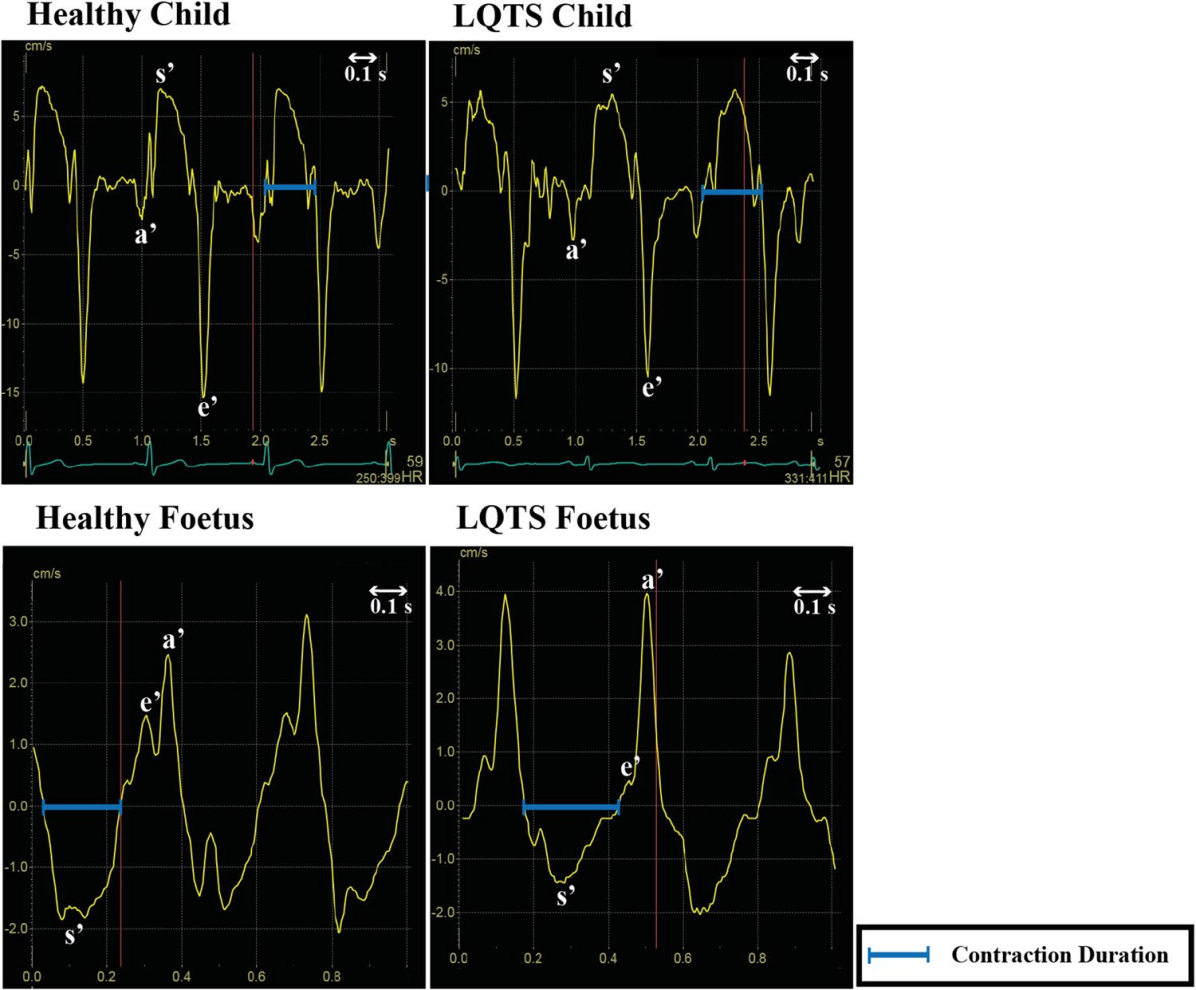
Myocardial contraction duration by cTDI in children (both upper panels) and in foetuses (both lower panels). Note that in the two lower panels the configuration of the velocity curves is inversed compared to the curves in children, due to a different position of the foetal heart with respect to the probe of the echocardiogram

The first echocardiogram of the LQTS foetuses was compared to the first echocardiogram of the controls (to avoid including multiple measurements per foetus). There was a longer CD_blinded_ in LQTS foetuses compared to controls with an absolute mean difference of 12 ms. The CL was also longer in LQTS patients compared to controls (∆10 ms). When the last available echocardiograms were included, there also was a longer CD_blinded_ in LQTS foetuses compared to controls (∆35 ms) and a longer CL (∆50 ms). Figure 4 shows an example of the difference in CD_blinded_ in foetal twins where one foetus was healthy and the other had LQTS type 3. Note that in the two right panels the configuration of the velocity curves is inverted compared to the curves in children, due to the vertex position of the foetuses.

## Discussion

### Main Findings

This study is the first to assess abnormal ventricular contraction patterns in paediatric LQTS patients using cTDI, and shows that both the contraction duration and the contraction duration blinded for a simultaneously recorded ECG assessed by cTDI can be used in the identification of LQTS in children. In addition, measurements of the contraction duration and the contraction duration blinded for a simultaneously recorded ECG have a strong correlation and agreement with the QT-interval on a 12-lead ECG and a high reproducibility. Moreover, it is feasible to measure the contraction duration blinded for a simultaneously recorded ECG in foetuses among a broad range of gestational ages making it a potential technique for future studies on the diagnostic value of the contraction duration blinded for a simultaneously recorded ECG for the identification of foetal LQTS.

### Mechanical Abnormalities in LQTS

Inhomogeneous prolongation of cardiac repolarization in LQTS patients [2] could impact on the regional mechanical cardiac function, since electrical and mechanical cardiac functions are inevitably connected via electro-mechanical coupling mechanisms [37]. Regional mechanical motion can be affected while parameters reflecting global function may still be in the normal range. High spatiotemporal resolution techniques are needed to detect such distinct alterations. With this study we showed a prolonged myocardial contraction duration in paediatric LQTS patients compared to age- and gender-matched controls using cTDI, which confirms the results of a recent study in paediatric patients by Brado et al [12] where a prolonged myocardial contraction duration was seen in paediatric LQTS patients using MRI. The prolongation of the myocardial contraction in our study is in approximately the same order of magnitude as described by Haugaa et al. [8] in adult LQTS patients using cTDI (about 40 ms) and had a concordant high inter- and intra-observer validity. None of the previous studies reported data on the diagnostic value of the presence of mechanical abnormalities.

Two cases have been reported regarding mechanical abnormalities in foetuses,[38, 39] using other modalities than cTDI (e.g. Pulsed Doppler). One of the cases was a LQTS type 3 foetus with sinus bradycardia, second-degree atrioventricular block and transient ventricular tachycardia, where a very short transmitral early deceleration time was measured by Pulsed Doppler echocardiography [38]. The authors suggested that recognition of a short mitral valve deceleration time may be helpful in the diagnosis of LQTS in the foetus. The other case was a 19-week-old LQTS type 1 foetus with a markedly prolonged left ventricular isovolumetric relaxation time on echocardiogram [39]. The measurements performed in these cases, were never validated in LQTS or correlated to QT-intervals. In a recently published multinational study, it was shown that the left ventricular isovolumetric relaxation time (LVIRT) is prolonged in LQTS foetuses, even when corrected for cycle length [40]. In our study we used a validated measurement for myocardial contraction duration, as a surrogate for QT-interval, and an approximation to the combination of the left ventricular isovolumetric relaxation time, the left ventricular contraction time and the LVIRT. LQTS foetuses seemed to have a longer myocardial contraction duration compared to controls. Unfortunately, we were not able to test this statistically due to the small sample size and unstructured collected data inherent in a pilot study.

### Underlying Mechanism of Mechanical Abnormalities in LQTS

It is postulated that a deranged calcium homeostasis may play a role in the underlying mechanism of this mechanical dysfunction in LQTS [3, 4, 6–8, 12, 41]. A prolonged QT-interval in LQTS implies a prolonged action potential duration (APD), and this may increase the calcium influx via L-type calcium channel and a concomitant increase in the calcium-induced calcium release from the sarcoplasmic reticulum. As a consequence the calcium concentration may increase in the cytoplasm, and the duration of calcium transient may be prolonged, which can cause an impaired diastolic relaxation and a prolonged contraction duration. The increased or sustained calcium concentration could be facilitated by early afterdepolarizations, not reaching threshold for the induction of action potentials, but causing an intracellular calcium distortion [3, 4, 7, 8, 11]. Otherwise, studies on LQTS type 3 patients suggest that mechanical abnormalities may be secondary to an increased intracellular sodium concentration caused by the persistent inward sodium current [10, 41, 42].

The inhomogeneous myocardial contraction is related to either an heterogeneous distribution of mid-myocardial cells (which generally have a longer APD) [8] or ion channels [11] throughout the myocardium, and reflect electrical dispersion [5, 10, 11].

### Clinical Implications and Translation to the Foetal Heart

Bearing in mind that in this study the LQTS children were mainly asymptomatic with not very long QTc-intervals, we highlight the potential of the myocardial contraction duration to detect even subtle mechanical abnormalities in paediatric LQTS patients. The myocardial contraction duration has therefore the ability to differentiate between controls and phenotype-negative LQTS children. We suggest that analysis of mechanical abnormalities might add additional information to the diagnosis in paediatric patients *suspected* of LQTS, used along with the QTc-interval on an ECG and provocation tests such as an exercise test or a brisk-standing test. In any case, the presence of mechanical abnormalities can be used in the risk stratification of paediatric LQTS patients as shown in previous studies [12].

The accuracy of diagnosing LQTS based on mechanical abnormalities is of a particular interest prenatally. As non-invasive tests for the diagnosis of LQTS prenatally are limited, and measuring the myocardial contraction duration in foetuses seems feasible, the contraction duration blinded for a simultaneously recorded ECG could potentially be used as a future diagnostic marker for LQTS. However, future studies should first focus on further validating measurements of the contraction duration blinded for a simultaneously recorded ECG in foetuses, and the establishment of normal ranges for healthy foetuses. Perhaps in the future, the contraction duration blinded for a simultaneously recorded ECG measured using cTDI could be used alongside the recognition of sinus bradycardia, second-degree atrioventricular block and ventricular arrhythmias in the diagnosis of foetal LQTS [26].

### Limitations

This study was limited to a small sample size. Previous studies showed age- and gender-related differences in QTc-interval, [43, 44] and although there are no data available about the influences of these factors on mechanical abnormalities, it is not inconceivable that the myocardial contraction duration can be influenced by age and/or gender. For this reason LQTS children were age- and gender-matched to controls. This resulted in a small sample size but strengthened the results of our study because matching removed the potential confounding effects of age and gender. Unfortunately, we were not able to specify the myocardial contraction duration based on genotype due to this small sample size.

As mentioned, only a limited number of symptomatic LQTS patients were included in the study, and although we performed a bootstrap analysis in order to assign the accuracy of myocardial contraction duration estimates, this group will be relatively underrepresented. We were therefore not able to analyse the presence of mechanical abnormalities as a risk factor for cardiac events. Contrariwise, we aimed to develop and validate an potential marker for diagnosing foetal LQTS, and were not interested in defining risk factors for cardiac events.

The slight difference in heart rate (although not statistically significant), and the presence of beta-blocker therapy in LQTS children might influence the myocardial contraction duration. Since there is no method to correct the myocardial contraction duration for heart rate, as there is for the QT-interval on a 12-lead ECG, we corrected using a multiple linear regression. Future experiments should elucidate the exact relationship between the CD and heart rate to establish an adequate correction formula. Most of the included patients were on beta-blocker therapy according to the guidelines, [13] which did not allow us to compare patients without beta-blocker therapy to controls. However, Haugaa et al. [8] showed that the prolonged contraction duration in LQTS was not attributable to the use of beta-blocker therapy.

## Conclusion

Myocardial contraction duration assessed by cTDI was prolonged in LQTS children and can be used in the identification of LQTS. Measuring the contraction duration in foetuses seems feasible, and a prolonged contraction duration may therefore be a potential marker for the prenatal diagnosis of LQTS in future. Further studies are required to support the measurement of the myocardial contraction duration as a diagnostic approach for foetal LQTS.

## Electronic supplementary material

Below is the link to the electronic supplementary material.

Supplementary material 1 (DOCX 42 KB)
