# Supplementary material for: A Potential Diagnostic Approach for Foetal Long-QT Syndrome, Developed and Validated in Children
Source: Pediatr Cardiol. 2018 May 22;39(7):1413–22. doi: 10.1007/s00246-018-1911-y (PMC6153877; doi:10.1007/s00246-018-1911-y)
Supplement: Supplementary file 1 — Supplementary material 1 (DOCX 42 KB) [file 246_2018_1911_MOESM1_ESM.docx]

Supplementary material

This supplementary material has been provided by the authors to give readers additional information about their work.

Supplementary Table

Supplementary Table 1. Description genotype LQTS-patients

| **Patient** | **Gender** | **Age (years)** | **Gene** | **Mutation** | **Nucleotide change** | **Coding effect** |
| --- | --- | --- | --- | --- | --- | --- |
| 1 | Male | 2 | KCNQ1 | p.(Ala344Val) | c.1031C>T | Missense |
| 2 | Male | 2 | SCN5A | p.(lle1768Val) | c.5302A>G | Missense |
| 3 | Male | 4 | KCNQ1 | p.(Val524Gly) | c.1571T>G | Missense |
| 4 | Male | 4 | KCNQ1 | p.(Arg591Cys) | c.1771C>T | Missense |
| 5 | Female | 14 | KCNQ1 | p.(Phe296Ser) | c.887T>C | Missense |
| 6 | Male | 7 | SCN5A | p.(lle1768Val) | c.5302A>G | Missense |
| 7 | Male | 10 | KCNQ1 | p.(Ala344Val) | c.1031C>T | Missense |
| 8 | Male | 11 | KCNQ1 | p.(Arg190Trp) | c.568C>T | Missense |
| 9 | Male | 11 | KCNQ1 | p.(Arg397Trp) | c.1189C>T | Missense |
| 10 | Male | 11 | KCNQ1 | p.(Phe296Ser) | c.887T>C | Missense |
| 11 | Male | 12 | KCNQ1 | p.(Ala344Val) | c.1031C>T | Missense |
| 12 | Female | 12 | KCNQ1 |  | c.1515-?_1590+?del | Deletion |
| 13 | Male | 14 | KCNH2 |  | g.150,648,803_150,652,487dup | Duplication |
| 14 | Male | 14 | KCNH2 | p.(Gly873Alafs*5) | c.2616del | Frameshift |
| 15 | Female | 14 | KCNH2 | p.(Tyr99Ser) | c.296A>C | Missense |
| 16 | Female | 14 | KCNH2 | p.(Tyr99Ser) | c.296A>C | Missense |
| 17 | Female | 15 | KCNQ1 | p.(Arg190Trp) | c.568C>T | Missense |
| 18 | Male | 15 | KCNQ1 | p.(Trp120Cys) | c.360G>C | Missense |
| 19 | Male | 14 | SCN5A | p.(lle1768Val) | c.5302A>G | Missense |
| 20 | Female | 15 | KCNH2 | p.(Ser906Leu) | c.2717C>T | Missense |
| 21 | Female | 17 | KCNH2 |  | g.150,648,803_150,652,487dup | Duplication |
| 22 | Male | 11 | KCNH2 | p.(Phe640Leu) | c.1920C>A | Missense |
| 23 | Male | 12 | KCNH2 | p.(Arg534Cys) | c.1600C>T | Missense |
| 24 | Female | 14 | KCNH2 | p.(Arg366*) | c.1096C>T | Nonsense |
| 25 | Female | 5 | SCN5A | p.(lle1768Val) | c.5302A>G | Missense |
| 26 | Female | 12 | KCNH2 | p.(Tyr99Ser) | c.296A>C | Missense |
| 27 | Female | 12 | KCNH2 |  | g.150,648,803_150,652,487dup | Duplication |
| 28 | Male | 9 | KCNH2 | p.( p.Trp927*) | c.2780G>A | Nonsense |
| 29 | Male | 9 | SCN5A | p.(Ile1768Val) | c.5302A>G | Missense |
| 30 | Male | 13 | KCNH2 | p.(Tyr99Ser) | c.296A>C | Missense |
| 31 | Male | 3 | KCNQ1 | p.(Arg190Trp) | c.568C>T | Missense |
| 32 | Male | 16 | KCNH2 | p.(Arg366*) | c.1096C>T | Nonsense |
| 33 | Male | 11 | KCNH2 | p.(Tyr99Ser) |  | Missense |
| 34 | Female | 14 | KCNH2 | p.(Gly873Alafs*5) | c.2616del | Frameshift |
| 35 | Female | 7 | SCN5A |  | 1795insD | In-frame insertion |
| 36 | Female | 3 | KCNQ1 | p.(Tyr184Ser) | c.551A>C | Missense |
| 37 | Male | 6 | KCNQ1 | p.(Val319Leu) | c.955G>C | Missense |
| 38 | Female | 4 | KCNQ1 | p.(Ala344Val) | c.1031C>T | Missense |
| 39 | Male | 0 | KCNQ1 | p.(Phe193Leu) | c.577T>C | Missense |
| 40 | Male | 0 | KCNH2 | p.(Arg582Cys) | c.1744C>T | Missense |
| 41 | Male | 7 | KCNH2 | p.(Arg534Cys) | c.1600C>T | Missense |

Supplementary Table

Supplementary Table 2. Description of the foetuses in the pilot study

| **PIN** | **Gender** | **GA** | **Case/Control** | **Gene** | **Mutation** | **Nucleotide change** | **Coding effect** | **CD_blinded_ in ms** | **CL**  **in ms** |
| --- | --- | --- | --- | --- | --- | --- | --- | --- | --- |
| 1 | Male | 24+1 | Control | NA | NA | NA | NA | 333 | 459 |
| 2 | Male | 17+4 | Control | NA | NA | NA | NA | 316 | 409 |
| 3 | Female | 17+4 | Control | NA | NA | NA | NA | 110 | 416 |
| 4 | Male | 20+3 | Control | NA | NA | NA | NA | 311 | 416 |
|  |  | 28+3 |  |  |  |  |  | 187 | 402 |
| 5 | Male | 34+1 | Control | NA | NA | NA | NA | 276 | 400 |
| 6 | Male | 17+6 | Case | KCNH2 | p.(Arg582Cys) | c.1744C>T | Missense | 244 | 449 |
| 7 | Female | 17+1 | Case | KCNH2 | p.(Arg252Glyfs*108) | c.754del | Frameshift | 288 | 421 |
|  |  | 21+1 |  |  |  |  |  | 309 | 432 |
|  |  | 29+1 |  |  |  |  |  | 329 | 496 |
| 8 | Male | 21+0 | Case | KCNQ1 | p.(Phe193Leu) | c.577T>C | Missense | 224 | 438 |
|  |  | 31+0 |  |  |  |  |  | 202 | 479 |
|  |  | 36+0 |  |  |  |  |  | 233 | 496 |
| 9 | Male | 19+4 | Case | SCN5A | p.(lle1768val) | c.5302A>G | Missense | 275 | 379 |
|  |  | 27+4 |  |  |  |  |  | 249 | 396 |
| 10 | Female | 22+0 | Control | NA | NA | NA | NA | 259 | 423 |
|  |  | 30+0 |  |  |  |  |  | 312 | 421 |
| 11 | Female | 19+2 | Case | SCN5A | p.(lle1768val) | c.5302A>G | Missense | 247 | 412 |
| 12 | Male | 31+6 | Case | SCN5A | p.(lle1768val) | c.5302A>G | Missense | 291 | 434 |
| 13 | Female | 22+1 | Control | NA | NA | NA | NA | 223 | 430 |
| 14 | Male | 32+3 | Case | KCNH2 | p.Glu698* | c.2092G>T | Nonsense | 336 | 555 |
|  |  | 29+3 |  |  |  |  |  | 309 | 522 |
|  |  | 23+3 |  |  |  |  |  | 267 | 440 |
| 15 | Male | 19+2 | Control | NA | NA | NA | NA | 172 | 364 |

PIN= Patient Identification Number, GA= Gestational age in weeks + days, NA= Not Applicable.
